# 3D Exoscope-Assisted Microvascular Anastomosis: An Evaluation on Latex Vessel Models

**DOI:** 10.3390/jcm9103373

**Published:** 2020-10-21

**Authors:** Valentina Pinto, Federico A. Giorgini, Maria Elisa Lozano Miralles, Achille Tarsitano, Maria Martina Panella, Riccardo Cipriani, Marco Pignatti

**Affiliations:** 1 Plastic Surgery, Azienda Ospedaliero-Universitaria di Bologna, via Albertoni 15, 40138 Bologna, Italy; valepinto@yahoo.it (V.P.); federico.giorgini3@gmail.com (F.A.G.); mariaelisa.lozano@gmail.com (M.E.L.M.); cipric1@yahoo.it (R.C.); 2 Plastic Surgery, Policlinico di Modena, University of Modena and Reggio Emilia, Largo del Pozzo, 71, 41125 Modena, Italy; 3 Oral and MaxilloFacial Surgery, Azienda Ospedaliero-Universitaria di Bologna, via Albertoni 15, 40138 Bologna, Italy; achille.tarsitano2@unibo.it; 4 DIBINEM (Dipartimento di Scienze Biomediche e NeuroMotorie), University of Bologna, 40126 Bologna, Italy; 5 Department of Economics and Management, Università di Parma, 43121 Parma, Italy; mmartinapanella@gmail.com; 6 DIMES (Dipartimento di Medicina Specialistica, Diagnostica e Sperimentale), University of Bologna, 40126 Bologna, Italy

**Keywords:** micro-anastomosis, exoscope, training

## Abstract

Background. Over the last few years, advances in technologies and digital imaging have led to the introduction of systems that enable a new approach to microsurgery and supermicrosurgery. The exoscope is a new magnification system that provides a 3D image of the surgical field: microsurgical procedures can be performed with the aid of this instrument. Here, we describe our preliminary experience with a high-definition 3D exoscope (VITOM^®^, Karl Storz, Tuttlingen, Germany), evaluating the characteristics of the instrument, and also its use as a magnification device for microanastomosis training. Methods. Six microsurgeons with various levels of experience were asked to perform three end-to-end anastomoses and two end-to-side anastomoses on latex vessel models, using, as a magnification system, the VITOM^®^ 3D 4K exoscope. None of the surgeons involved had previous experience with the exoscope, with robotic surgery, with endoscopic surgery, nor with training simulators. Results. The results of the reported evaluation of the tool’s qualities, (VITOM Quality Assessment Tool) included: a good focusing of the surgical field; high image quality; strong luminance; good magnification; clear stereoscopy; and excellent freedom of movement. The exoscope proved to be user-friendly. A constant reduction in the time needed to perform the microsurgical anastomosis at each exercise was recorded. Among other advantages were the easy switching from the magnified image to the macroscopic view, superior ergonomics allowing a relaxed posture while performing the anastomosis, adequate space, and a convenient setting for the assistants to view the operating field. Conclusions. Our study showed that the exoscope VITOM 3D can be successfully used as a magnification tool for microsurgical anastomosis on synthetic vessels, and that it can also be helpful during training courses in microsurgery.

## 1. Introduction

Since 1960, microsurgery has revolutionized the world of reconstructive surgery, by allowing surgeons to perform sophisticated procedures, with the use of surgical loupes and operating microscopes that magnify the surgical field. Over the last few years, advances in technologies and digital imaging have led to the introduction of robotic systems such as the “Da Vinci” robot, which allowed a new approach to microsurgery and supermicrosurgery. 

More recently, the exoscope, a new magnification system that provides a 3D image of the surgical field, has become available. The image on the screen can be magnified as much as with a microscope, therefore microsurgical procedures can be conducted with the aid of this instrument. 

The aim of this report is to evaluate the exoscope as a magnification tool in microsurgical vessel anastomosis, both for experienced surgeons and for the training of those with less experience. 

In addition, we evaluated this new device and its advantages in terms of image quality, ease of setting, and comfort for the surgeon.

According to this preliminary experience, the high-definition 3D exoscope (VITOM^®^) may also be considered for microanastomosis training. Gaining experience in the technique of vascular anastomosis on vessels of a caliber of only a few millimeters requires practice. The basic microsurgical courses usually consist of some theoretical instructions, followed by several hours of practical exercises under the microscope. As an example, microsurgical courses, to be approved by the Italian Society of Microsurgery (SIM), must provide at least twenty hours of hands-on training [1]. The first microsurgical exercises aim to teach depth perception, stereoscopic vision, and dexterity under the microscope magnification [2].

The next step is teaching the performance of end-to-end anastomosis using microsurgical instruments, usually on a 2 mm or 3 mm synthetic tube under the microscope, and with an 8–0 or 9–0 suture. Reports on the use of exoscopes for neurosurgical interventions have started to appear [3], although only a few papers have described the applications for microsurgical vessel anastomoses during free flaps reconstruction [4,5].

## 2. Materials and Methods

### 2.1. VITOM^®^ 3D 4K Exoscope 

**Figure 1:**
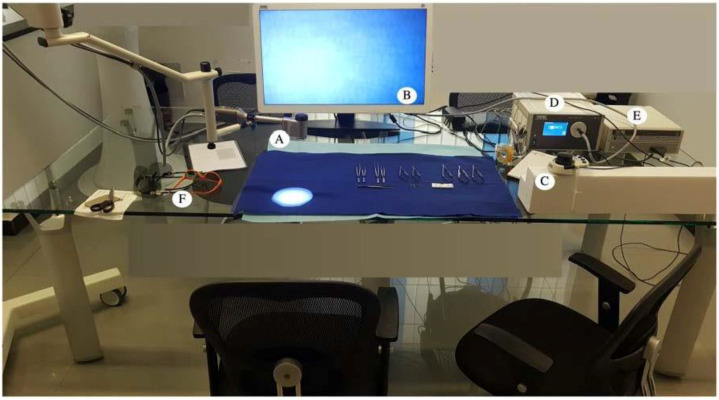
The study setting. The microsurgical instrumentation and the complete exoscope set are visible. The latter includes: (**A**) the two compact and light cameras coupled at the tip of a small crane; (**B**) a three-dimensional 4k high-resolution video display; (**C**) a control joy-stick (Pilot) to zoom, focus and finely adjust the camera position; (**D**) the light-source; (**E**) the computer hardware; (**F**) several pairs of three-dimensional eyeglasses.

The exoscope is a device able to display the images recorded by two compact and light cameras, coupled at the tip of a small crane, on a 3-dimensional 4 k high-resolution video screen. The complete exoscope set also includes a control joystick (Pilot) to zoom, focus, and finely adjust the camera position, and several pairs of three-dimensional eyeglasses (Figure 1).

### 2.2. Study Design

Six microsurgeons with various levels of experience participated in the study. All participants filled out a form, which included demographic data (age, gender), hand dominance (right-handed, left-handed, ambidextrous), previous experience (including traditional conventional microsurgery, robotic surgery and simulators, endoscopic surgery and simulators, video games experience), and the level of training (resident, research fellow, junior consultant, senior consultant).

An official representative of the device’s manufacturer explained, in 15 min, how to set up and use the system and its various components. 

**Figure 2:**
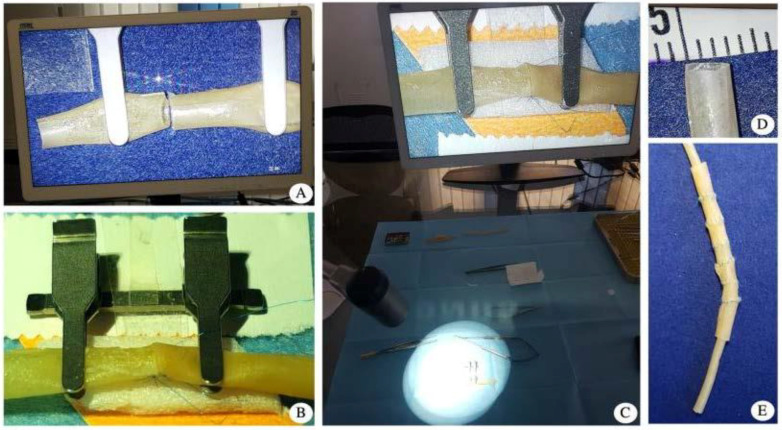
(**A**) magnified screen view (without the 3D glasses) of the two stumps of the synthetic 2 mm vessel held in position by an Ackland clamp; (**B**) naked-eye appearance of the end-to-end anastomosis setting at the end of the back-wall anastomosis; (**C**) picture taken through the 3D glasses: the screen view at the end of the back-wall anastomosis. The real field is also in view. The surgeon can easily see both views by tilting their head; (**D**) measurement of the vessel; (**E**) the synthetic vessel after several anastomoses have been performed to optimize resources during microsurgical anastomosis training.

**Figure 3:**
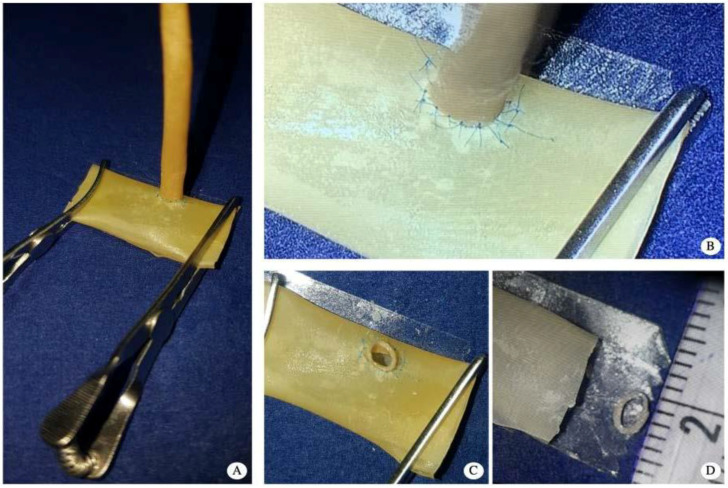
(**A**) naked-eye appearance of the end-to-side anastomosis setting at the end of the exercise; (**B**) picture taken through the 3D glasses: screen view at the end of the anastomosis; (**C**) naked-eye appearance of the anastomotic site at the patency check; (**D**) caliber of the two vessels used for end-to-side anastomosis.

During the study, each surgeon used the exoscope VITOM 3D as a magnification system for the first time. They were asked to perform three end-to-end anastomoses (3 mm caliber double layer latex vessels) (Figure 2) and two end-to-side anastomoses (2.8 mm caliber double layer latex vessel to 9 mm caliber single layer latex vessel) on latex vessel models that were fixed on a foam platform and secured with a double-opposing Ackland vascular clamp (Figure 3).

The microvascular anastomoses were performed with an interrupted suture technique, using a 9.0 nylon thread. At the end of the test, each participant reported their experience on a 4-point Likert scale through the VITOM Quality Assessment Tool [6], a questionnaire assessing a subjective evaluation of the tool’s qualities. Finally, the time needed to complete each microanastomosis by each surgeon, the number of stitches used, and the quality of the anastomosis, provided an objective evaluation of the performance.

## 3. Results

The surgeons’ demographic data are reported in Table 1.

The mean age was 40.6 years, and the range was from 28 years to 65 years old. Four participants were male and two were female.

Five participants were right-handed, one was left-handed.

In terms of previous microsurgical experience, the surgeons were classified as follows: one senior consultant boasted vast microsurgical expertise (category D: over 30 years of practice); two testers were competent (category C: microsurgeons who usually perform this kind of surgery, but have less than 20 years of experience), one participant was considered a beginner (category B: surgeon that attends courses but does not routinely perform microsurgical procedures); two were plastic surgeons in training (residents) and this was their first experience with microsurgical vessel anastomosis (category A: no previous experience). 

None of the surgeons involved had previous experience with the exoscope, with robotic surgery, with endoscopic surgery, nor with their training simulators. Fifty percent of the enrolled surgeons (*n* = 3) had minimal experience with videogames.

All of the surgeons involved in the study completed the five required microsurgical anastomoses.

### Qualities of the Tool

The results of the reported evaluation of the tool’s qualities, (VITOM Quality Assessment Tool) are listed in Table 2. 

The image quality provided by the exoscope was considered superior to that of the operating microscope, as were the operating field luminance and magnification, as rated by the three surgeons with previous microsurgical experience (value 4).

Stereoscopy was considered good (value 3) by 50% of the testers (*n* = 3) and very good (value 4) by the remaining 50%. 

The magnification rate was considered to be very good. However, unsatisfactory depth perception at high magnification, causing an occasional empty grasp, was reported by four of the surgeons (shown as extra comments.). 

Luminance was unanimously considered to be very good.

The focusing of the surgical field was judged as very good (value 4, continuously in focus) by four of the participants, while two of them judged these characteristics as good (value 3).

Two surgeons referred to a minimal eye strain (value 3); four to no strain (value 4)

Three surgeons considered the device to be extremely user-friendly concerning maneuverability (value 4); the remaining three judged it to be good (value 3). 

**Figure 4:**
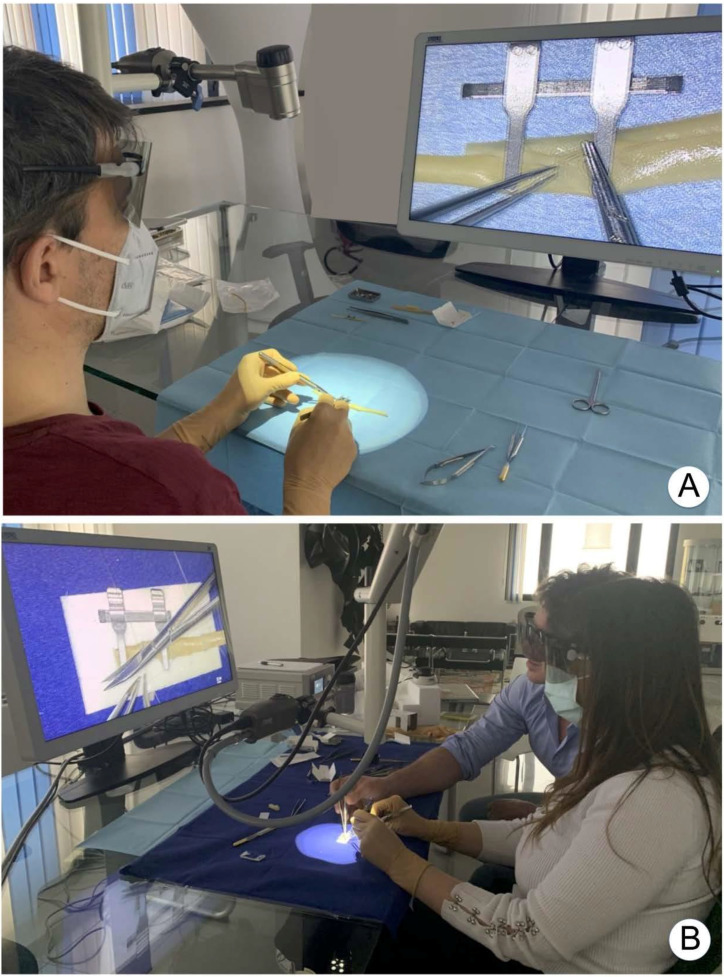
Posture and freedom of movement of the surgeons is shown. (**A**). Single operator (**B**). Possibility for two operators, during a microsurgical course to sit next to each other and share the same view.

The three surgeons with previous microscope experience declared that the new device granted greater freedom of movement, made it possible to switch easily from the magnified image to the macroscopic view of the anastomotic site, and favored a more relaxed posture of head, neck, and trunk during the procedure (Figure 4).

None of the surgeons complained of physical discomfort in relation to posture, especially of the neck, and all six described the posture as natural (value 4).

The overall performance improved significantly for each participant during the five microanastomoses sessions. 

**Figure 5:**
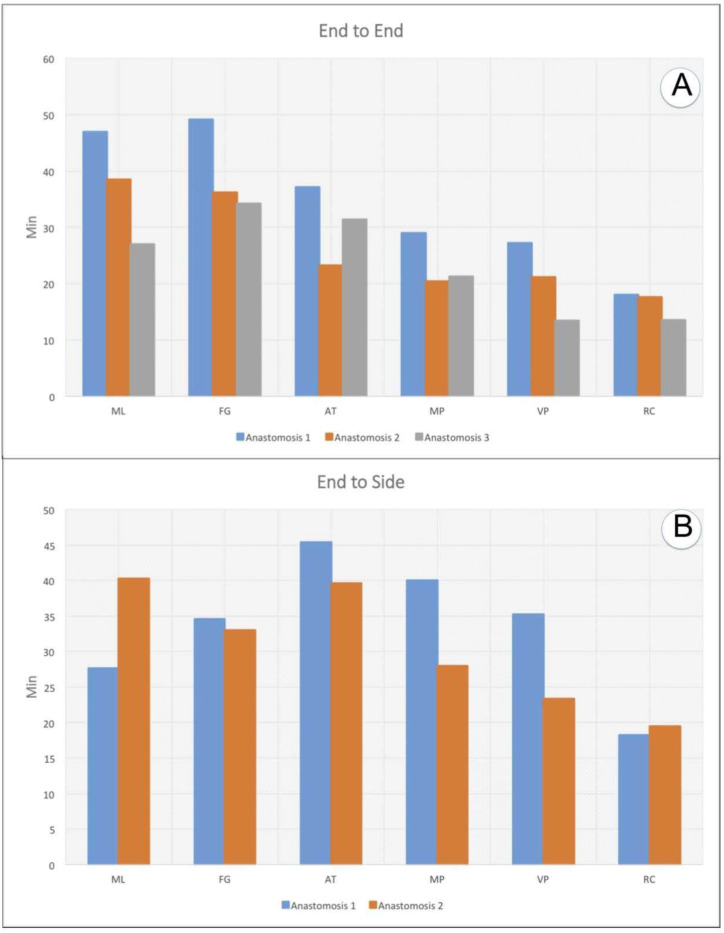
Charts showing the improvement of surgical performance with practice: (**A**). Time spent by each surgeon to complete the three end-to-end anastomoses and (**B**) Time spent to complete the two end-to-side anastomoses.

Operative time decreased for each surgeon, with an initial phase of technical adaptation, followed by rapid improvement of technical skills and confidence (Figure 5).

## 4. Discussion

The introduction by Nylean in 1954 of the operating microscope into clinical practice [7,8] marked a milestone in the history of microsurgery. In 1960, Jacobson and Suarez performed the first vascular anastomosis using an operating microscope [9].

### 4.1. Efficiency of The Exoscope as a Magnification Device

The use of the exoscope in neurosurgery has been reported to result in better ergonomics and accessibility of the surgical field.

Belykh et al. anastomosed, with microsurgical techniques and the aid of the exoscope magnification, the small vessels of humans, bovines, and rats [10].

They concluded that the VITOM 3D 4k was superior to the microscope for clarity and sharpness, the size of surgical field, and depth perception, without lengthening the operating time when compared to the microscope. 

Similarly, Ahmad et al., when comparing the 3D exoscope with the conventional microscope during free flap surgery for flap dissection and microvascular anastomosis, concluded that the exoscope is an effective microsurgical tool that may be considered as an alternative to conventional optical magnification [11]. 

Piatkowski et al. performed a bilateral breast reconstruction with free Deep Inferior Epigastric Perforator (DIEP) flaps using for internal mammary vessels dissection and microvascular anastomosis the 3D exoscope on one side, and the conventional microscope on the contralateral side. They concluded that the exoscope did not reach the performance of a good quality microscope [4].

Grammatica et al. used a 3D 4K exoscopic system in a clinical setting for head and neck microsurgical reconstruction. They concluded that the harvesting of free flaps and microanastomosis with the 3D 4K exoscopic system was feasible [12]. 

De Virgilio et al. performed a clinical human study of free flap microvascular anastomosis using the VITOM 3D exoscope in 10 consecutive patients undergoing reconstruction after ablative surgery for head and neck carcinoma. Microvascular anastomoses were performed successfully using the exoscope in all patients, without any need for a conventional microscope [13]. 

In our study we also found that the instrumentation provided adequate magnification to perform the anastomosis of artificial vessels of 2 mm to 3 mm of caliber. Moreover, its use is easy to learn and the performance of the surgical task improves rapidly, as demonstrated by the reduction in time needed for each anastomosis. A limit of our study in this aspect was the low number of anastomoses performed and the bias of the artificial setting, however the trend to a fast, progressive improvement at each anastomosis can clearly be seen.

### 4.2. The Exoscope as a Training Device

Laboratories dedicated to teaching technical aspects of the new technologies to surgeons in training are frequently being organized. The exoscope-assisted technology grants a shorter learning curve compared to other magnification systems, and not requiring a change of posture improves the speed with which the anastomosis can be performed. 

A preliminary study demonstrated the usefulness of the three-dimensional exoscope when teaching some basic tasks requiring high magnification, although in in this work no microsurgical vessel anastomoses were performed [6].

In our study, we tested the VITOM 3D as a magnification device for microsurgical anastomosis. We believe that, as a training model, synthetic vessels have several advantages compared to animal tissues [14], therefore we chose to use small latex tubes. One additional advantage of the exoscope, particularly important in a teaching setting, is the position of the 3D monitor that allows one or more assistants to stand near the surgeon and observe the procedure from a similar angle. (Figure 4b). In a clinical setting two or more monitors can be used, in different positions, to allow the operators to stand one in front of the other, or in the most comfortable and useful position.

Based on our experience, the exoscope can be considered to be an excellent device to provide magnification during microsurgical courses. Other training models for microsurgical anastomoses that take advantage of modern technologies have been previously proposed [15]. Using the exoscope from the initial microsurgical training will allow the surgeons to become acquainted with a device that they will later use when practicing in a clinical setting.

### 4.3. Ergonomics

At present, standard microsurgical reconstruction requires the use of the operating microscope with the surgeon positioned in proximity to the ocular lenses, remaining in a posture uncomfortable for the shoulders, neck, and wrists, especially during long procedures. 

In the last few years, more attention has been paid to the physical well-being of surgeons during long interventions [5].

The wide field of view and deep focusing attributes of the exoscope enable its positioning above the surgical field, and minimize the need for re-positioning and re-focusing during the procedure. Moreover, the freedom of movement of the head and neck of the surgeon without losing the magnified view allows a more relaxed posture, and lets the surgeon change positions and relax their muscles (Figure 4).

### 4.4. Cost

No economic evaluation on the use of the VITOM-3D exoscope has been conducted [6].

A precise evaluation of costs in our setting is difficult due to the method of purchasing goods in public hospitals, although an estimate could be attempted. The cost of a modern, high-quality operating microscope routinely used in microsurgery varies from 130,000 to 400,000 euros [3] while that of the VITOM^®^ 3D 4K exoscope averages 150,000 euros.

One advantage of the exoscope in a large university hospital is also related to the possible adjunctive use of some components of this equipment in other kind of surgeries, such as endoscopic procedures, or the entire exoscope in other surgical specialties that may need surgical field magnification or a better view in narrow spaces [3]. This could result in a faster depreciation of the investment for the healthcare organisation.

## 5. Conclusions

We tested the use of the exoscope VITOM 3D for microsurgical anastomosis on synthetic vessels as a model for training courses in microsurgery, and to evaluate its qualities as a magnification tool. 

According to the surgeons that tested it in this study, these are some of the qualities of the device:

• Excellent image quality and depth perception
• Possibility to easily switch from the magnified image to the macroscopic view of the anastomotic site
• Available space for the assistants to view the operating field, right at the surgeon’s side, demonstrating a clear advantage for education
• Easy to use and become acquainted with the apparatus, as shown by the rapid decrease in the time needed to complete each anastomosis
• Other device characteristics, as compared to the usual surgical microscope:
• Greater freedom of movement
• Superior ergonomics which permitted a relaxed posture of the head, neck, and trunk of the surgeon while performing the anastomosis.

The main limit of our study was the small number of surgeons who tested the instrument, and the small number of anastomoses performed. The artificial vessels used were 2 mm and 3 mm wide, and results may be different in living tissues and vessels of 1 mm. 

The evaluations of the surgeons involved in this study were comparing the exoscope to their previous, routine practical use of the surgical microscope in clinical practice. A direct comparison of the exoscope to a surgical microscope, side by side, on the same tasks, could be useful in the next study. 

Further experience can be gained and described as soon as the device becomes more widely available. 

## Figures and Tables

**Table 1:** The surgeons’ demographic data are reported. **Level of Experience: A** Novice, **B** advances beginner, **C** Competent, **D** expert. **Level of Training: SC:** Senior Consultant. **JC**: Junior Consultant. **R**: Resident.

3D Exoscope-Assisted Microvascular Anastomosis						
**Participant**	1	2	3	4	5	6
**Gender**	M	F	M	F	M	M
**Age**	46	39	65	28	30	38
**Hand Dominance**	Right	Right	Right	Right	Left	Right
**Previous Experience**						
**Level of Training**	SC	JC	SC	R	R	SC
**Traditional Conventional Microsurgery**	C	C	D	A	A	B
**Robotic Surgery**	A	A	A	A	A	A
**Simulator**	A	A	A	A	A	A
**Endoscopic Surgery**	A	A	A	A	A	C
**Simulator**	A	A	A	A	A	B
**Video Games**	B	A	A	A	B	B

**Table 2:** The results of the reported evaluation of the tool’s qualities, (VITOM Quality Assessment Tool) are listed.

Vitom Quality Assessment Tool				
	Not Acceptable 1	Acceptable 2	Good 3	Very Good 4
Image Quality				100% (*n* = 6)
Stereoscopic Effect			50% (*n* = 3)	50% (*n* = 3)
Magnification Rate				100% (*n* = 6)
Luminance				100% (*n* = 6)
Focusing			33.3% (*n* = 2)	66.7% (*n* = 4)
Eye Strain			33.3% (*n* = 2)	66.7% (*n* = 4)
Maneuverability			50% (*n* = 3)	50% (*n* = 3)
Exercises are Possible in a Natural Posture				100% (*n* = 6)
There is a Wide Working Space				100% (*n* = 6)
Overall			33,3% (*n* = 2)	66.7% (*n* = 4)
Extra Comments depth perception at high magnification	66.7% (*n* = 4)		33.3% (*n* = 2)	
